# Durability of Two Epoxy Adhesive BFRP Joints Dipped in Seawater under High Temperature Environment

**DOI:** 10.3390/polym15153232

**Published:** 2023-07-29

**Authors:** Ruitao Niu, Yang Yang, Zhen Liu, Ziyang Ding, Han Peng, Yisa Fan

**Affiliations:** 1 School of Aerospace Engineering, Zhengzhou University of Aeronautics, Zhengzhou 450046, China; niu.ruitao@163.com; 2 Institute of Mechanical Engineering, Materials and Transportation, Peter the Great Saint-Petersburg Polytechnic University, Saint-Petersburg 195251, Russia; y691483@163.com; 3 School of Mechanical Engineering, North China University of Water Resources and Electric Power, Zhengzhou 450045, China; lz17335585447@163.com (Z.L.); 19138123694@163.com (Z.D.); penghan@ncwu.edu.cn (H.P.)

**Keywords:** adhesive joint, salt dipping solution, durability, failure mechanism

## Abstract

Fiber-reinforced polymers (FRPs) have great potential in shipbuilding. As a new type of material, basalt-fiber-reinforced polymer (BFRP) has received increasing attention due to its good economic and environmental performance. In this paper, BFRP single-lap joints (SLJs) bonded by Araldite^®^2011 and Araldite^®^2014 were selected as sample objects, the joints, aged for 240 h, 480 h, and 720 h, were experimentally analyzed in 3.5% NaCl solution/5% NaCl solution at 80 °C. The sequential dual Fickian (SDF) model was used to fit the water absorption process of the dumbbell specimen material. By comparison, the water absorption of the material occurred mainly on the adhesive and the water absorption of Araldite^®^2011 was higher than that of Araldite^®^2014. The decrease in the T_g_ of the aged joint adhesive was characterized by DSC, and the TG test showed that the polymer material in the joint was degraded by the damp–heat effect. The quasi-static tensile test showed that the decrease in joint failure strength was positively correlated with the water content of the solution. The Araldite^®^2011 adhesive joint showed better mechanical properties and stability than the Araldite^®^2014 adhesive joint, while the secondary crosslinking of the bound water with the polymer chain resulted in a slight increase in the stiffness of the aged joint. From comprehensive observation of the macro-section and SEM-EDX images, it is concluded that the failure mode of the joint changes from fiber tearing to mixed failure of fiber tearing and adhesive layer cohesion, and the plasticizing effect of the epoxy resin in the adhesive and chemical corrosion of salt ions weakens the adhesive layer’s bond strength.

## 1. Introduction

The application of fiber-reinforced polymers (FRPs) is one of the key directions in automotive, shipbuilding, construction, aerospace, and other fields nowadays. FRP materials have the advantages of being lightweight, having a high strength, providing good insulation, corrosion resistant, being designable, and so on, providing innovative ideas for the selection of materials in various fields [1,2,3]. Currently, widely used FRP materials include carbon-fiber-reinforced plastics (CFRPs), glass-fiber-reinforced plastics (GFRPs), basalt-fiber-reinforced plastics (BFRPs), aramid-fiber-reinforced plastics (AFRPs), etc. [4,5,6]. Compared with other fiber-reinforced materials, the advantage of BFRPs is that they not only have the characteristics of fiber-reinforced composites, excellent mechanical properties, strong durability, strong corrosion resistance, and low hygroscopicity, but also have excellent recyclability, environmental protection, and lower cost [7]. The basalt fiber is made from molten basalt rock, and its manufacturing process is similar to glass fiber but with less energy consumption and no additives [8]. However, the connection technology between FRP and other materials limits its further development in the field of engineering. Some traditional connection methods such as riveting, bolting, and welding are not suitable for FRP. These methods often result in sticky wall damage and fiber breakage, and inevitably stress concentration problems [9,10], so the life of the joint may be much lower than expected. In particular, fiber-reinforced composite materials are anisotropic and difficult to connect with other metal materials, which has become a major challenge for the application of composite materials. Therefore, in many cases, we give priority to the interconnection of the same composite materials. As a new type of connection, adhesive bonding has the advantages of uniform stress distribution, good fatigue performance, and being lightweight [11]. It is also more adaptable to composite materials, because both matrix and adhesive have certain polymer properties [12,13]. In addition, compared with ordinary mechanical connection, bonding has a larger bearing area and better fatigue resistance [14]. Bonding can also impart some ductility to the joint [15], which is currently a widely recognized connection method for composites. Chen et al. [16] studied the quasi-static tensile properties of CFRP-Al joints in bonding, riveting, and mixed joints. The results showed that compared with riveted joints, the peak load value was significantly increased, the energy absorption level was improved, and the failure prevention ability was enhanced.

FRP material has attracted the interest of researchers in the shipbuilding, offshore oil, and marine infrastructure industries due to its excellent mechanical properties [17]. From an economic point of view, the low availability and high life cycle cost of high-quality wood are the main limitations of wooden boats. Hull superstructures made of aluminum alloy have poor performance at high temperatures and are prone to severe fatigue cracking, resulting in expensive repair costs. However, FRP materials are lighter (10% and 36% lighter than aluminum or steel ships of similar size, respectively), have lower maintenance costs and fuel consumption, and the life cycle cost is about 7% lower than similar steel ships [18]. These also provide sufficient motivation for the application of FRP materials in the shipbuilding industry. Composite materials were first used in shipbuilding by the U.S. Navy in the 1940s, and designers have given more and more attention to the use of composite materials since then. Now, whether it is civilian speedboats, yachts, small- and medium-sized commercial ships, military patrol boats, or warships (part of warship superstructure), the shipbuilding industry is using a large number of composite materials. Kevlar-fiber-reinforced composites have been used in the cruisers to enhance the resistance to high-speed impact. Glass/carbon-fiber-reinforced composite (PVC) cores are also used in hull structures due to their high stiffness and strength and the high shear properties of foam cores. In the coming years, the use of composite materials in ships will continue to grow and become more versatile, including use in decks, bulkheads, propellers, propulsion shafts, pipes, pumps, and other mechanical parts [19]. The service environment of ships is more complex and variable than that on land, with common erosion environmental conditions including temperature, humidity, UV, corrosion, load, etc. [20]. The multifactor coupling is a great test of the comprehensive properties of composite bonded joints. In order to ensure the stability and safety of the structure, it is necessary to study the performance of the joints under various limit conditions.

Due to long-term exposure to the sea surface, the temperature of hulls can reach 60–80 °C, and the temperature can reach more than 50 °C in a limited space. Temperatures of 80 °C and a high humidity environment have a great influence on the bonding properties of composite joints, especially on the mechanical properties and fatigue resistance of composite materials, and will produce many adverse factors [21]. Zhang et al. [22,23] studied the failure load after aging of single-lap joints under conditions of 80 °C/40% relative humidity and 80 °C/90% relative humidity. The test results showed that the greater the humidity is under high temperatures, the more obvious the decline in the failure load. Avendaño et al. [24] selected an acrylic adhesive to bond one-way CFRPs with biopolymers; the joint was tested at −30, 23, and 80 °C. The results showed that the strength of the joint was significantly reduced due to the softening of the binder. Banea and daSilva [25] studied the performance of adhesives for automotive epoxies at room temperature (RT), −40, and 80 °C through a large number of samples. The experimental results show that the strength of the adhesive decreases obviously at high temperature. By contrast, as the temperature drops, the adhesive becomes more brittle and exhibits a higher tensile strength. Yao et al. [26] found that the binding strength of BFRP-steel increased to some extent in the temperature range of −25 °C to 50 °C, but decreased significantly in the temperature range of 50 °C to 100 °C. They also concluded that high temperatures weaken the interfacial bond between the BFRP and binder.

Moisture is also a basic environmental factor affecting the joint performance of composite materials, especially the moisture–heat coupling [27]. The coupling effect of water and temperature (hydrothermal effect) is usually more harmful than the effect of a single factor [28]. The degradation of the mechanical properties of joints is always accompanied by physical and chemical damage of composites, microcracking and debonding in the matrix. Furthermore, the fiber–matrix interface and interlaminar delamination are all related to water/heat [29,30,31], while chemical damage in the matrix is caused by the hydrolysis reaction of the substrate and interface, which weakens the interface bonding and interlaminar adhesion [32,33,34]. The effect of moisture on FRP adhesive joints depends on several parameters, including the material in the joint, bonding method, degree of curing, surface treatment process, and exposure conditions [35]. In the joints, the polymer matrix and adhesive of FRP are the most affected by water, both of which may undergo plasticization, expansion, and hydrolysis. These mechanisms can reduce the glass transition temperature (T_g_) and the elastic modulus and strength [15]. Moisture can also affect the stability of interfacial adhesion, and the effect of interfacial degradation on joint durability is even more serious than that of adhesive materials or adhesives. [36]. In addition, water absorption also affects the failure mode of the joint, resulting in cohesion failure to interface failure, which is due to the weakening of molecular energy, resulting in irreversible changes. However, some physical damage caused by moisture, such as plasticization, has been found to be somewhat reversible after drying [37]. Banea et al. [38] studied the effect of water absorption on the mechanical behavior of structural adhesives modified with thermal expansion particles (TEPs). The results show that the tensile properties, strength, and elastic modulus decrease with the increase in water content, and recover after desorption at the same temperature as the initial environmental conditions.

Ships operating at sea place higher demands on the durability of composite bonded joints. After prolonged immersion in seawater, the chemical expansion caused by chloride ion corrosion will increase the porosity between the resin matrix and the fiber, and corrodes the bonding interface of the joint, which will eventually lead to debonding [39]. It has also been pointed out that the osmotic effect of salt ions usually leads to a decrease in the chemical activity of aqueous solutions. The crosslinking behavior of the polymer forms a semi-permeable membrane, which hinders the movement of water molecules and large inorganic ions, while deionized water does not contain solute ions, its molecules can diffuse faster, so brine is generally less aggressive than deionized water [37,38,39,40]. In addition, there are differences in seawater durability among various fiber composites [41,42,43]. In order to more accurately understand the life cycle of FRP materials in seawater environments, scientists have conducted research. Wang et al. [44] used basalt fiber CBF13-1200 and epoxy resin 9804 A/B as materials to simulate the marine environment by configuring salt solution. The samples were immersed in salt solution at room temperature of 25 °C for 1, 3, and 6 weeks. The damage evolution and fracture shape during fatigue loading were recorded by electron microscope. The results show that under fatigue load, salt water causes obvious damage to the static strength of the BFRP and the interface deteriorates with aging time; the fatigue life prediction results show that the fatigue strength of BFRP decreases after salt water corrosion. Sheng Li et al. [39] summarized the quasi-static tensile properties and dynamic properties of FRP composites exposed to simulated seawater and a seawater sand concrete environment. The results show that the degradation degree of FRP composites increases with the increase in exposure time, temperature, stress level, and alkalinity/salinity of the dipping solution. Based on a study of the dynamic degradation of glass/basalt fiber-reinforced polymer (GFRP/BFRP) composites after seawater exposure and strain rate sensitivity under dynamic loading, it was found that the impact strength, Young’s modulus, and fatigue life were all reduced by seawater exposure. Wang Y L et al. [45] derived the bilinear bond stress–slip relationship for different exposure periods according to the bending test results, based on these bond stress–slip relations, the full range of shear stress behavior along the bond length and debonding load can be obtained analytically. The experimental and numerical results show that the maximum shear stress at the FRP–concrete interface tends to move backward with the extension of exposure time, indicating that the dry–wet circulation of the salt solution weakens the mechanical properties of the FRP–concrete interface. Zhongyu Lu et al. [46] found, from seawater and tap water immersion, that bare BFRP tendons exhibited higher degradation rates in seawater than in tap water due to the coupling of water molecules and chloride ions.

It can be seen from the above studies that high temperatures and salt solutions have different forms of influence on the mechanical properties and aging failure of joints. When the two factors work together, the effects do not simply add up. In order to further understand the change in mechanical properties and aging failure mode of composite joints soaked in salt solution at high temperature, the authors selected BFRP-BFRP single-overlap joints bonded by two adhesives with different properties: ductile epoxy adhesive Araldite^®^2011 and brittle epoxy adhesive Araldite^®^2014, under the accelerated aging condition of 80 °C, and two dipping environments of 3.5% NaCl solution and 5% NaCl solution. The thermal and mechanical properties of each joint were tested after aging for 240 h, 480 h, and 720 h. The variation in the joint properties under different aging conditions was analyzed. The failure morphology of each joint was observed and the failure mechanism of the joints summarized. Currently, there are few available test data on BFRP-BFRP joints, and BFRP-adhesive-bonded joints may have BFRP delamination and fiber fracture; the failure mode is more complex than that of metal bonding joints [47,48]. The durability of BFRP-adhesive-bonded joints in seawater has not been fully clarified, the purpose of this paper is to study the durability and failure mechanism of BFRP-adhesive-bonded joints in the seawater dipping environment, and to provide data support for the application of BFRP in the shipbuilding industry.

## 2. Experimental Process

### 2.1. Materials

The fiber cloth laying direction in the BFRP panel used in the experiment is [0/90/0/90/0/90], and the fiber cloth is made of epoxy resin matrix filled with basalt fibers processed through prepreg processing. The selected properties of the BFRP fiber cloth are shown in Table 1. According to the standard ASTMD586801 [49], the geometric size of the BFRP is 100 mm × 25 mm × 2 mm. Two adhesives with different properties were selected for the experiment: Araldite^®^2011 and Araldite^®^2014 (Huntsman Advanced Materials Co., Ltd. (The Woodlands, TX, USA); 2011 and 2014 for short). Araldite^®^2011 is a multi-purpose, two-component, room temperature curing paste adhesive with high strength, low shrinkage, good toughness, and good dynamic load resistance, it is the standard diglycidyl ether of bisphenol-A with average molecular weight <700 together with an amine hardener. Araldite^®^2014 is a thixotropic and environmental-corrosion-resistant brittle epoxy adhesive cured at room temperature. Its chemical composition is isopropyl diphenol, formaldehyde, bis (2,3 epoxypropyl) butane, bisphenol a-epoxy resin, malonyldiacrylate, and hydroquinone [50]. Selected properties of the Araldite^®^2011 and Araldite^®^2014 adhesives are shown in Table 2.

### 2.2. Single-Lap Joint Design

This paper studies the aging failure of BFRP-BFRP single-lap joints in different environments. In practice, the simplest and most effective single-lap joint specimens need to be considered in the following aspects: (1) Only the deformation in the shear direction is considered. (2) Peel stresses of the eccentric load are ignored in the shear test. (3) The shear stress (*τ*) of the adhesive is constant at the overlap length (*L*), which can be calculated from the following equation, where *P* is the force applied and b is the width of the joint [51]:

(1) $$\tau =\frac{P}{bL}$$

**Figure 1:**
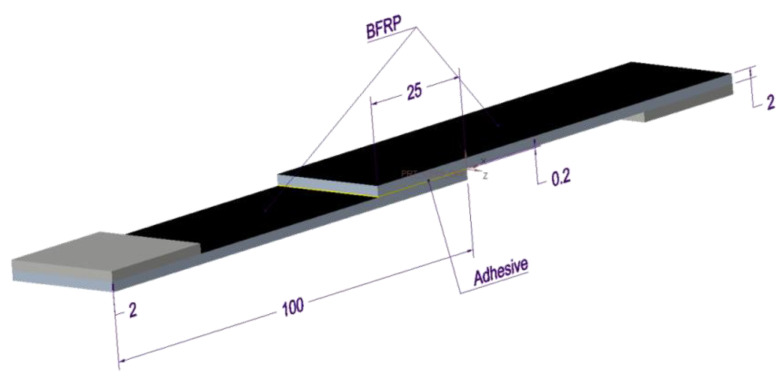
Configurational geometry and dimensions (mm).

The specific size and three-dimensional model of the BFRP-BFRP single-lap joint specimen prepared by the experiment are shown in Figure 1. It is proved that the geometrical parameters of the bonding area play a key role in the performance of the joint. To achieve the best performance of the adhesive-bonded joint, the length and width of the lap joint are 25 mm and the thickness of the adhesive layer is 0.2 mm. To prevent the substrate surface adhesion from affecting the joint performance, it is necessary to perform surface treatment on the substrate [52,53]. The surface treatment method of acetone cleaning was used in this experiment. The production standard of the bonding joint was according to ISO4587:2003 [54].

### 2.3. Experimental Setups

Araldite^®^2011 and Araldite^®^2014 were used as the adhesives in this experiment. Each adhesive was placed in 80 °C/3.5% NaCl solution and 80 °C/5% NaCl solution, with serial numbers a–d. Each environment was divided into three groups according to aging time, 240 h, 480 h, and 720 h, and a control group without aging treatment. The groups were numbered 1–4 according to the aging time, and 3 specimens in each group were selected for treatment.

**Figure 2:**
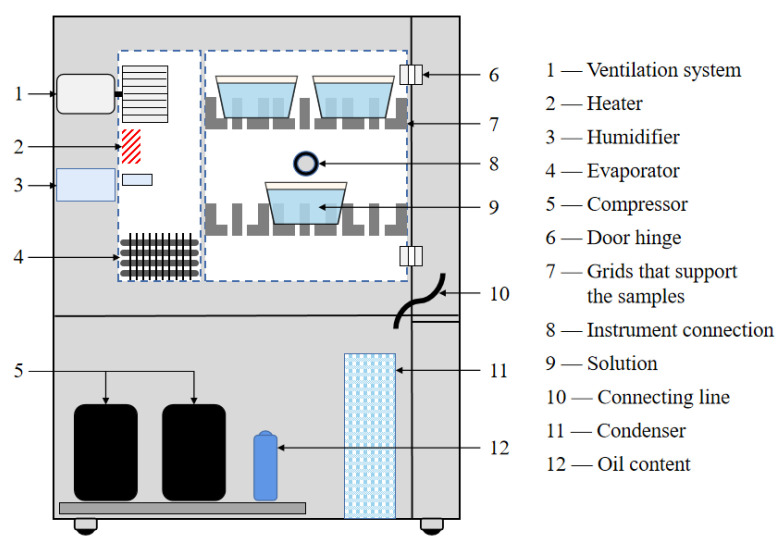
Environmental cabinet principle model.

The BFRP-BFRP single-lap joint meeting the experimental standard was put into a drying experimental box to remove the moisture absorbed by the adhesive layer during the adhesive joint curing process. After drying, the joints that needed to be aged were put into pre-prepared deionized water, 3.5% or 5% salt solution in groups, and finally put into the environment cabinet (WS-1000 of the Weiss Equipment Experimental Company; the principle model is shown in Figure 2). After reaching the predetermined ageing time, the joint was removed and left to dry at room temperature for 8 h.

### 2.4. Specific Test Methods

**Figure 3:**
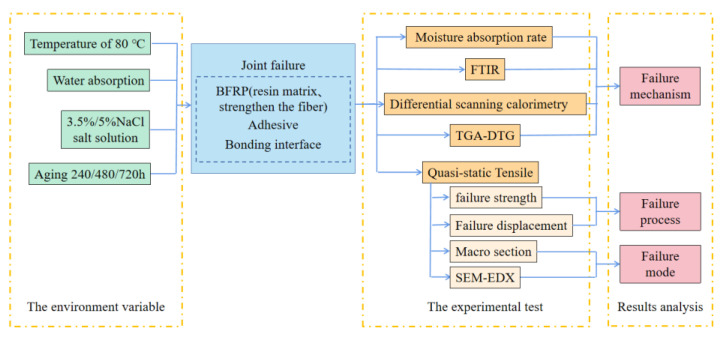
Diagram of experimental principle.

After the aging experiment is completed, it is necessary to test the residual performance of the joint, explore the degradation mechanism of the joints bonded by different adhesives in various environments, characterize and analyze the cross-section, and summarize the failure process and failure mode of the joints. The design of the test method is shown in Figure 3.

#### 2.4.1. Water Absorption Test

**Figure 4:**
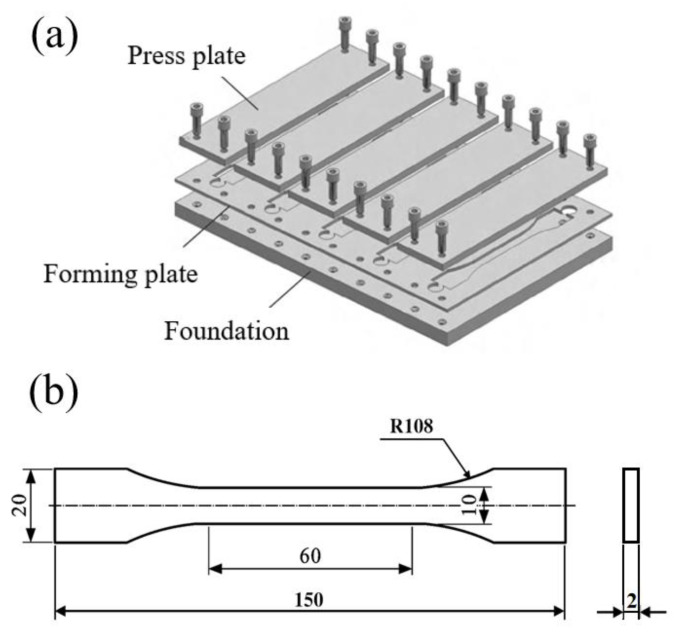
(**a**) The mold of dumbbell specimen is made. (**b**) Dumbbell specimen geometry and dimensions, in mm.

In order to more clearly and intuitively analyze the role of moisture in joint aging, dumbbell-shaped standard parts were prepared using two types of adhesives and BFRP panels for water absorption testing. A two-component adhesive gun was used to fill the adhesive evenly in the mold, and care was taken to avoid bubbles in the dumbbell-type specimen during the filling process; the mold and geometric parameters of the dumbbell-type specimen are shown in Figure 4. Three specimens were used from each group, and then the obtained data were averaged. The water absorption rate was calculated by measuring the weight of each group of specimens at an interval of 24 h and comparing the initial mass. An analytical balance with an accuracy of 0.1 mg was used for the weight measurement. Before the weight measurement, the surface moisture was wiped off with absorbent paper, and the weight measurement process did not exceed 30 min to avoid the influence of the external environment on the experimental results. The formula for the water absorption rate is given by Equation (2).

(2) $$M_{t}=\frac{W_{t}-W_{0}}{W_{0}}\times 100\%$$

where *M_t_* represents the water absorption rate at time *t*, *W_t_* (mg) represents the mass at time *t*, and *W*_0_ (mg) represents the original mass.

#### 2.4.2. Differential Scanning Calorimetry

The glass transition temperature (T_g_) is an inherent property of polymer materials such as adhesives and BFRP. T_g_ depends on many factors such as molecular weight, chemical crosslinking, plasticizer, length of polymerization chain, and functional groups [55], and its change is also one of the main factors leading to the deterioration of joint performance. In order to more accurately describe the role of T_g_ in joint aging, a differential scanning calorimeter (Mettler Toledo, DSC3+, Greifensee, Switzerland) was used for DSC analysis of the adhesives’ T_g_ at different aging points.

The test was conducted in a nitrogen atmosphere with a temperature rise/drop rate of 5 °C/min and a temperature range from −70 °C to 200 °C. The adhesive sample, with a mass of about 5 mg, was taken from the failure surface of the adhesive-bonded joint. Each test requires two heating processes, the first to remove the thermal history of the sample, and the T_g_ is determined from the second heating process [56].

#### 2.4.3. Thermogravimetric Analysis—Differential Thermogravimetry

Non-isothermal thermogravimetric analysis (TGA) was used to measure the thermogravimetric curve of the weight loss change of the adhesive at the temperature controlled using the program at a constant rate of change. The sample weight loss rate, St, corresponding to different temperatures was analyzed. The calculation formula is shown in Equation (3). At the same time the relationship between the sample weight loss rate and temperature was obtained by using differential thermogravimetric analysis (DTG), and the thermal stability of the two adhesives under the unaged and two aging conditions was compared.

(3) $$S_{t}=\frac{W_{0}-W_{t}}{W_{0}}\times 100\%$$

The thermogravimetric analysis was performed in purged nitrogen at room temperature (RT) −800 °C at a heating rate of 5 °C/min, with sample weights of 35–45 mg.

#### 2.4.4. Quasi-Static Tensile Test

**Figure 5:**
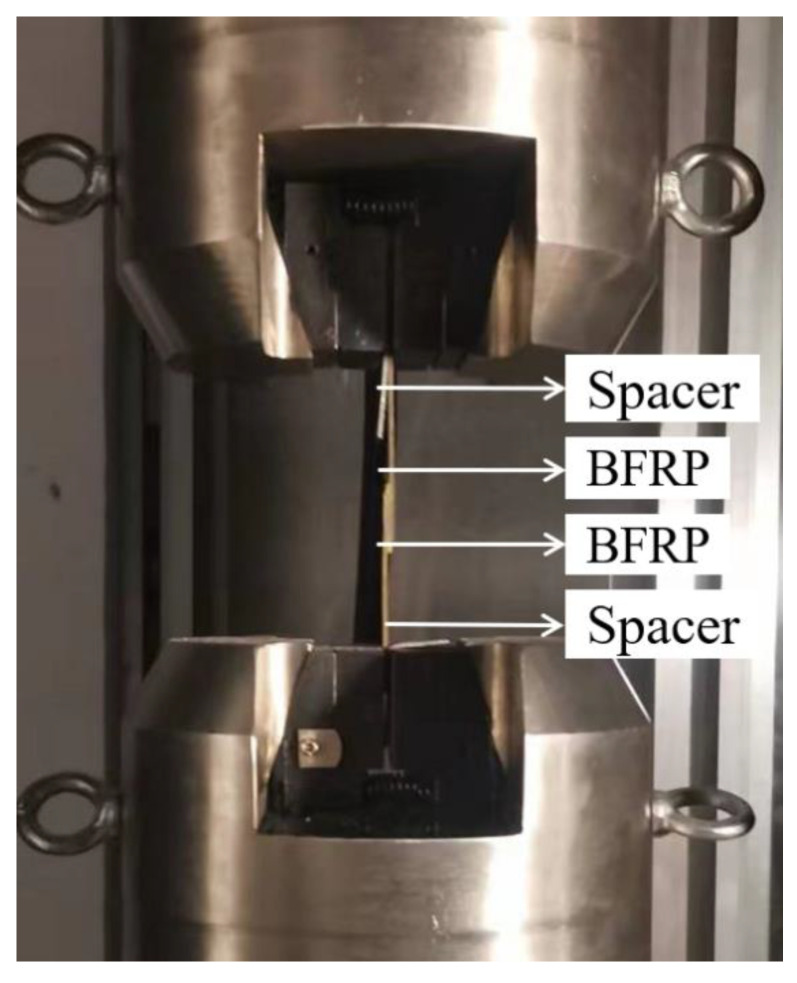
Quasi-static tensile test.

The quasi-static tensile test was performed using a universal tensile testing machine (JXYB305C, Xinguang Testing Machine Manufacturing Co., Ltd., Jinan, China, 300 KN), and the tensile speed was 2 mm/min. A 2 mm thick gasket was clamped at both ends of the specimen to eliminate the bending stress in the tensile process. In order to ensure the authenticity and reliability of the experimental results, the experimental data obtained were averaged. The average failure strength of each specimen can be read by the control computer connected to the tensile machine; the quasi-static tensile test is shown in Figure 5. After processing the obtained data, the average failure strength curve and load–displacement curve of the two kinds of joints in the unaged and three environments can be drawn, and the effect of the aging environment and aging time on the mechanical properties of the joints can be concluded by analyzing the curves’ changes.

## 3. Results and Analysis

### 3.1. Moisture Absorption Rate of Dumbbell Specimens

Due to the anisotropy of the sample materials, the unsteady diffusion of water roughly follows Fick’s second law, which can be simplified as:

(4) $$\frac{\partial C}{\partial t}=\frac{\partial }{\partial x}(D_{x}\frac{\partial C}{\partial x})+\frac{\partial }{\partial y}(D_{y}\frac{\partial C}{\partial y})+\frac{\partial }{\partial z}(D_{z}\frac{\partial C}{\partial z})$$

where *D* is the diffusion coefficient, *C* is the concentration of the diffused substance (component), *t* is time, and *x*, *y*, and z are the spatial coordinate axes of the concentration gradient along the three directions. Assuming that the sample thickness is in the *x* direction, and the size in the other two directions is much larger than this direction, so it can be idealized as *y*→∞, *z*→∞, and water undergoes one-dimensional diffusion in the *x* direction [57]:

(5) $$\frac{\partial C}{\partial t}=D\frac{\partial^{2}C}{\partial x^{2}}$$

**Figure 6:**
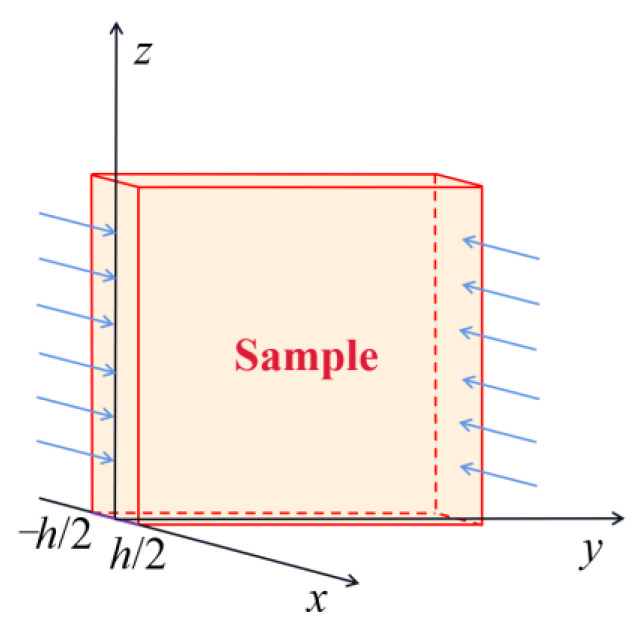
Diffusion in large plane sheet sample space.

Let the sample thickness be *h* (Figure 6). The initial concentration of water in the uniform sample is $C_{0}$ when *t* = 0. The boundary condition is that when *t* ≥ 0, the two surfaces of the sample are at a constant water concentration $C_{\infty }$, and the initial conditions and boundary conditions can be written as Equation (6):

(6) $$\{\begin{array}{c} C_{0}-h/2\;<\;x\;<\;h/2\;t=0\\ {\;C}_{\infty }\;x=-h/2,\;x=h/2\;t\geq 0 \end{array}$$

By combining the above conditions with the diffusion (Equation (5)), the moisture concentration distribution of the sample at any position and time in the *x* direction can be expressed as Equation (7):

(7) $$\frac{C(x,t)-C_{0}}{C_{\infty }-C_{0}}=1-\frac{4}{\pi }\sum_{n=0}^{\infty }\frac{{\left(-1\right)}^{n}}{\left(2n+1\right)}exp\left[-\frac{{\left(2n+1\right)}^{2}\pi^{2}D}{h^{2}}t\right]cos\frac{(2n+1)\pi x}{h}$$

The water absorption of the sample at time *t* is the integral of the water concentration at each x position:

(8) $$M_{t}=\int_{-h/2}^{h/2}\left(C-C_{0}\right)dx=\int_{-h/2}^{h/2}Cdx$$

The initial water concentration in the sample is generally ideally considered as $C_{0}=0$, and the final results are as follows:

(9) $$\frac{M_{t}-M_{0}}{M_{\infty }-M_{0}}=1-\frac{8}{\pi^{2}}\sum_{n=0}^{\infty }\frac{1}{{\left(2n+1\right)}^{2}}exp\left[-\frac{{\left(2n+1\right)}^{2}\pi^{2}D}{h^{2}}t\right]$$

$M_{0}$ is the initial moisture content in the material and $M_{\infty }$ is the saturated moisture content; so:

(10) $$\frac{M_{t}}{M_{\infty }}=1-\frac{8}{\pi^{2}}\sum_{n=0}^{\infty }\frac{1}{{\left(2n+1\right)}^{2}}exp\left[-\frac{{\left(2n+1\right)}^{2}\pi^{2}D}{h^{2}}t\right]$$

$M_{t}$ is measured experimentally and is usually expressed in the form of a percentage, as shown in Equation (1). The diffusion coefficient, *D*, can be obtained from Equation (11):

(11) $$D=\frac{\pi }{16}{\left(\frac{h}{M_{\infty }}\right)}^{2}{\left[\frac{M(t_{1})-M(t_{2})}{\sqrt{t_{1}}-\sqrt{t_{2}}}\right]}^{2}$$

However, through fitting attempts, it is found that the fitting degree of Fick’s second law is higher in the nearly linear part at the initial stage of diffusion. However, in the middle and later period, due to changes in the external environment, hygroscopic curves show great deviations, which is known as non-Fickian hygroscopicity. The initial stage of diffusion is divided into two stages. At the end of the first stage, water absorption enters a pseudo-equilibrium state, which is also the beginning of the second stage. In order to better fit the water absorption of the second stage, the sequential dual Fickian (SDF) model was adopted [58]:

(12) $$\begin{array}{l} C(x,t)=\left[1-\frac{4}{\pi }\sum_{n=0}^{\infty }\frac{{\left(-1\right)}^{n}}{2n+1}\exp\left(-\frac{{\left(2n+1\right)}^{2}\pi^{2}D_{1}}{h^{2}}t\right)\cos\frac{(2h+1)\pi x}{h}\right]C_{1\infty }+\\ \phi (t-t_{d})\left[1-\frac{4}{\pi }\sum_{n=0}^{\infty }\frac{{\left(-1\right)}^{n}}{2n+1}\exp\left(-\frac{{\left(2n+1\right)}^{2}\pi^{2}D_{2}}{h^{2}}(t-t_{d})\right)\cos\frac{(2h+1)\pi x}{h}\right]C_{2\infty } \end{array}$$

The two-part Fickian model is considered to be a parallel mechanism, the water concentration is equal to the boundary concentration when the sample is saturated, while $C_{t}=C_{1\infty }\;$+$\;C_{2\infty }$, $D_{1}$, and $D_{2}$ are diffusion coefficients of the two-part mechanism respectively, *t_d_* is the time of the transition from the first stage to the second stage, and φ(t) is the step function to complete the transition:

(13) $$\phi (t-t_{d})\{\begin{array}{c} {0\;t<t}_{d}\\ 1\;t\;\geq t_{d} \end{array}$$

The value of the step function indicates that diffusion enters the second stage as long as the time $t\;\geq \;t_{d}$, otherwise the second mechanism will not play a role and the integration of spatial variables can be obtained as follows:

(14) $$\begin{array}{l} M_{t}=\left[1-\frac{8}{\pi^{2}}\sum_{n=0}^{\infty }\frac{1}{{\left(2n+1\right)}^{2}}\exp\left(-\frac{{\left(2n+1\right)}^{2}\pi^{2}D_{1}}{h^{2}}t\right)\right]M_{1\infty }+\\ \phi (t-t_{d})\left[1-\frac{8}{\pi^{2}}\sum_{n=0}^{\infty }\frac{1}{{\left(2n+1\right)}^{2}}\exp\left(-\frac{{\left(2n+1\right)}^{2}\pi^{2}D_{2}}{h^{2}}(t-t_{d})\right)\right]M_{2\infty } \end{array}$$

The saturated water absorption rate of the second stage $M_{\infty }={\;M}_{1\infty }$+$M_{2\infty }$, $M_{t}$ is still determined by experiment.

**Figure 7:**
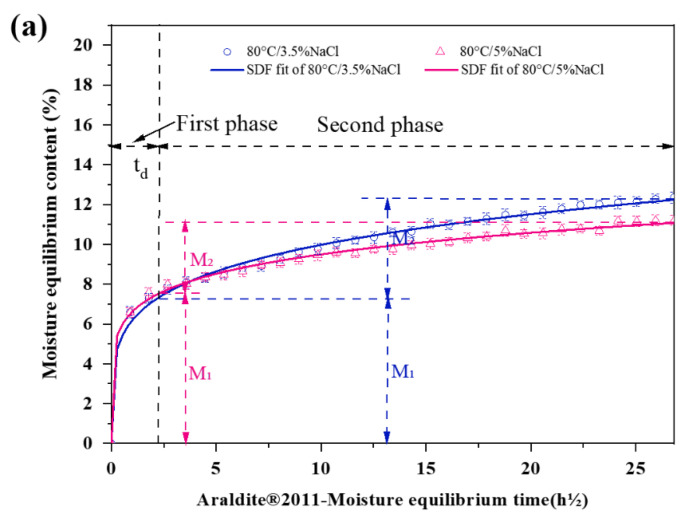

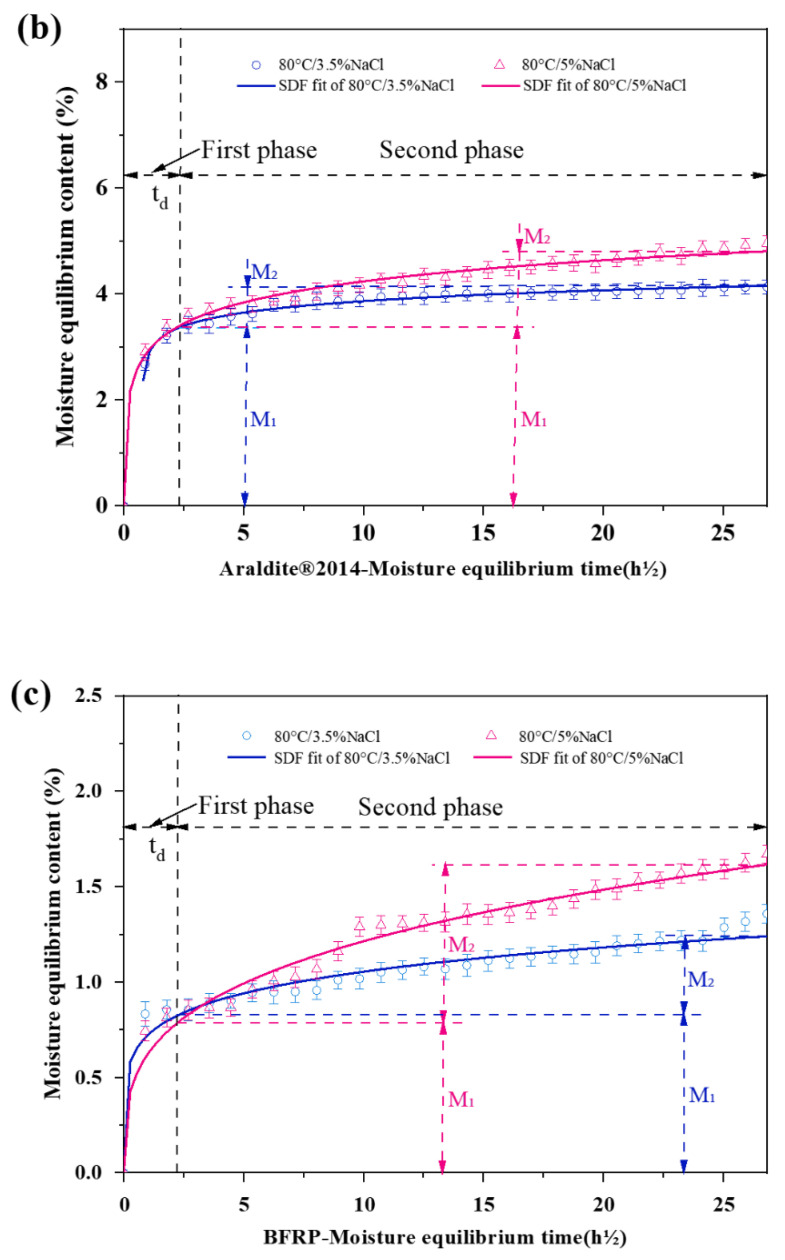
Fitting curves of water absorption rate of specimen material by sequential dual Fickian model: (**a**) Araldite^®^2011 adhesive, (**b**) Araldite^®^2014 adhesive, (**c**) BFRP.

Figure 7 was obtained by fitting the experimental water absorption data with the sequential dual Fickian law. It can be seen from the figure that the SDF model can well fit the water absorption of the joint throughout the ageing process. Water absorption increases with aging in both environments. The speed of water absorption, i.e., the slope of the curve, decreases rapidly from the initial maximum value; most of the water absorbed by the material is in the first 240 h, and the material tends to be saturated in the middle and late period. According to the definition of the dual Fickian model, we select the moment when the slope changes significantly to divide the water absorption process into two different stages. The water absorption speed in the first stage is very fast, and the absorption of water is mainly due to the diffusion filling of adhesives, the resin matrix, pores, and voids between the fiber and matrix layers [59]. High temperatures can accelerate the diffusion of water, and the resulting thermal expansion further increases the porosity. In the second stage, the diffusive water absorption of the material is close to saturation, but there is still a certain degree of water absorption, indicating that there are other water absorption mechanisms of the material: on the one hand, the corrosion of salt ions increases the porosity, on the other hand, the combination of the material’s hydrophilic groups with water molecules. There is little difference in water absorption between the two salt solutions, and the difference is mainly reflected in the second stage by comparing *M*_1_ and *M*_2_. As can be seen from (b) and (c), the water absorption increases slightly with the increase in salt concentration, which is due to the chemical expansion caused by excess chloride ion corrosion, which can promote the enlargement of resin pores and de-bonding between the matrix and fibers [39].

**Figure 8:**
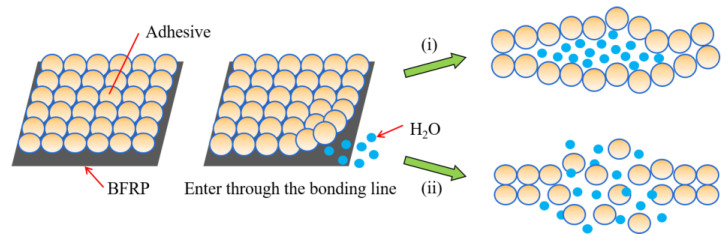
Schematic diagram of the adhesive hydrolysis mechanism.

By comparing the three panels of Figure 7, it can be seen that the water absorption rate of the adhesive in the joint is much higher than that of the BFRP plate, so it can be preliminarily judged that the performance degradation caused by water mainly occurs in the adhesive. The irreversible effects on adhesives on the molecular level are mainly manifested in two aspects (Figure 8) [60]:

(i) Chemical degradation leads to an increase in the internal pores of the entangled polymer chain, promoting chain expansion, and eventually forming microcracks in the polymer network.

(ii) The water molecules interact with the polar groups of the resin, and the absorbed water continually collides with the crosslinked chains in the adhesive, resulting in chain breakage and segment leaching. Similar cases have been reported in the literature.

The water absorption rate of the Araldite^®^2011 adhesive is higher than that of the Araldite^®^2014 adhesive, indicating that the Araldite^®^2011 adhesive, as a ductile adhesive, is more hydrophilic than the brittle Araldite^®^2014 adhesive, which may be related to the functional groups in the molecular chain [58]. Because water molecules fill the free volume, the reaction with functional groups is a key factor in causing the material to absorb more water.

### 3.2. DSC Analysis

**Figure 9:**
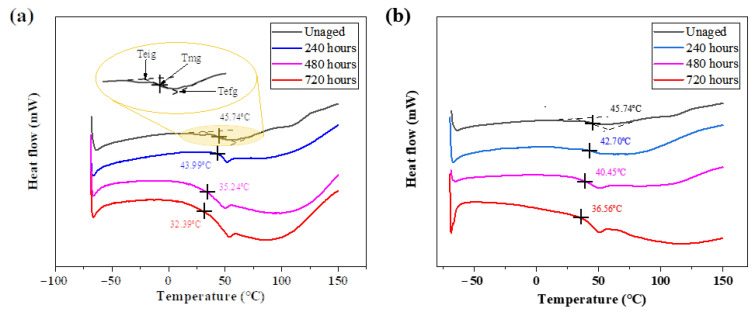

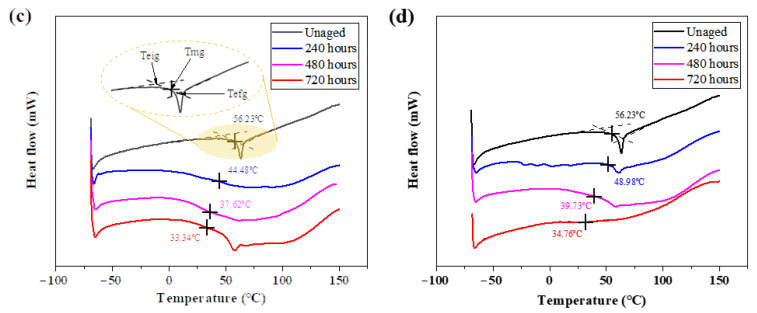
DSC test curves of two adhesive samples: (**a**) 2011 adhesive aged for 0/240/480/720 h in 3.5% NaCl environment; (**b**) 2011 adhesive aged for 0/240/480/720 h in 5% NaCl environment; (**c**) 2014 adhesive aged for 0/240/480/720 h in 3.5% NaCl environment; (**d**) 2014 adhesive aged for 0/240/480/720 h in 5% NaCl environment.

The glass transition temperature is an important parameter to determine the properties of adhesives and BFRP composites. Three temperatures were measured to determine the T_g_ of the two adhesives [61]: (i) the epitaxial starting temperature, T_eig_; (ii) the midpoint temperature, T_mg_; and (iii) the epitaxial termination temperature, T_efg_. According to the ASTM-D3418 standard [62], T_mg_ has more practical significance and is designated as T_g_ in a wide range of applications. Therefore, this paper chooses the T_mg_ value as the reference value of T_g_ change, and the process of determining T_mg_ is shown in Figure 9a,c. It can be seen from the figure that the T_g_ of the two types of adhesive in each environment decreased slightly with the aging increase. On the one hand, long-term exposure to high humidity will cause a plasticizing effect and chemical modification of the adhesive and resin matrix, thus reducing the T_g_ [61]. On the other hand, T_g_ is related to the density of the crosslinked polymer chain, and a humid and hot environment may lead to local molecular chain fracture and segment leaching, meaning the crosslinked density may decrease, leading to a decrease in T_g_ [63].

Comparing the two environments after aging for 720 h: the T_g_ of the Araldite^®^2011 adhesive decreased by 9.76 °C in an 80 °C/3.5% NaCl solution environment and 9.18 °C in an 80 °C/5% NaCl solution environment; the T_g_ of the Araldite^®^2014 adhesive decreased by 22.89 °C in an 80 °C/3.5% NaCl solution environment and 21.47 °C in an 80 °C/5% NaCl solution environment. It can be seen that the decline rate of the two salt solutions is similar, corresponding to the result of similar water absorption. Among the two kinds of adhesives, the water absorption rate of Araldite^®^2011 is higher than that of Araldite^®^2014, but the reduction in T_g_ is smaller, because the water absorption analysis shows that water is absorbed by the polymer in two different ways [64]: (a) as free water, it occupies the free space of the polymer, leading to plasticization of the material, which can make T_g_ drop; (b) as binding water, it can form hydrogen bonds with hydrophilic groups such as hydroxyl and amine in the polymer network, which can increase T_g_. In the water absorption test, it has been found that the hydrophilicity of the Araldite^®^2011 adhesive is stronger than that of the Araldite^®^2014 adhesive, indicating that the Araldite^®^2011 adhesive has more hydrophilic groups. This means that the second absorption effect will be more pronounced, weakening the overall downward trend in T_g_.

### 3.3. TGA-DTG Test

**Figure 10:**
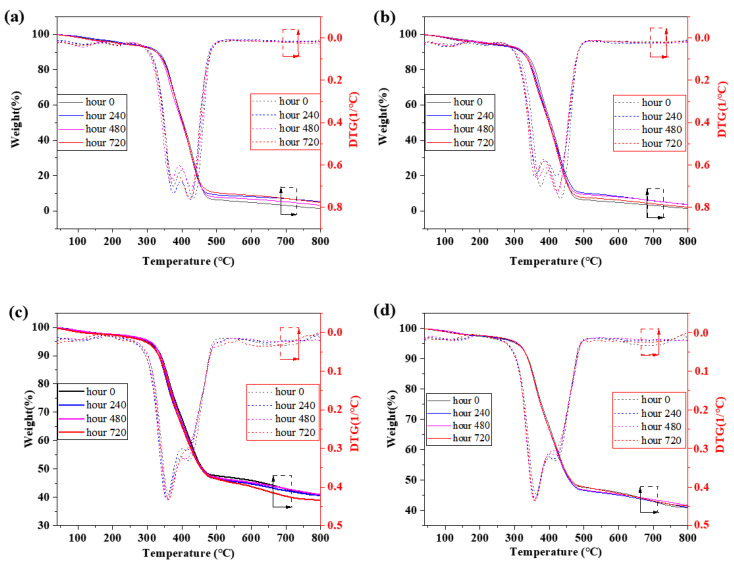
TGA-DTG test curves of two adhesive samples: (**a**) 2011 adhesive aged for 0/240/480/720 h in 3.5% NaCl environment; (**b**) 2011 adhesive aged for 0/240/480/720 h in 5% NaCl environment; (**c**) 2014 adhesive aged for 0/240/480/720 h in 3.5% NaCl environment; (**d**) 2014 adhesive aged for 0/240/480/720 h in 5% NaCl environment.

TGA curves can well reflect the changes in thermal stability of the two adhesives with temperature, but their deficiency is that they cannot accurately measure the weight loss range, weight loss speed, and the corresponding temperature of some less significant weight loss. However, all these are clearly expressed in TGA’s differential curve DTG, as shown in Figure 10.

It can be noted that the TGA-DTG curves of different aging times almost overlap, and the curves of the two environments also show great similarity, indicating that the aging time and salt solution environment cause no obvious changes in the thermal stability of the adhesive. In the thermogravimetric curve, the weight of the samples all showed a “step-like” decline, and the main weight loss interval was in the range of 240–500 °C, which is due to the polymer properties of the adhesive [65]. In this temperature range, materials are pyrolyzed, releasing compounds such as carbon dioxide, isocyanate, ammonia, cyanogen hydroxide, and carbon monoxide. [66]. Analysis of the DTG curve reveals that there is a less obvious weight loss peak around 130 °C, which may be related to water loss and the release of volatile compounds [63]. Comparing the weight loss rate of the two adhesives in this area, the Araldite^®^2011 adhesive is slightly higher than the Araldite^®^2014 adhesive. It can be seen from the previous analysis that the water absorption rate of the Araldite^®^2011 adhesive is higher than that of the Araldite^®^2014 adhesive. The main weight loss interval, weight loss peak temperature, and residual rate of each curve are shown in Table 3. In the main weight loss interval, the weight loss of both adhesives showed two stages, the maximum weight loss of the Araldite^®^2011 adhesive was in the second stage, and that of the Araldite^®^2014 adhesive was in the first stage. After 600 °C, the weight loss velocity of the Araldite^®^2011 adhesive tends to stabilize, but the weight loss velocity of the Araldite^®^2014 adhesive has a decreasing tendency after a fluctuation, in which the weight loss rate of the samples aged for 720 h even decreases to almost zero, as shown in Figure 10c,d. When the temperature reaches 800 °C, the maximum residual rate of the Araldite^®^2011 adhesive is 5.35%, while that of the Araldite^®^2014 adhesive can reach 41.72%. From the above differences, we can conclude that the thermal stability of the Araldite^®^2014 adhesive is significantly stronger than that of the Araldite^®^2011 adhesive.

### 3.4. Joint Failure Strength Analysis

**Figure 11:**
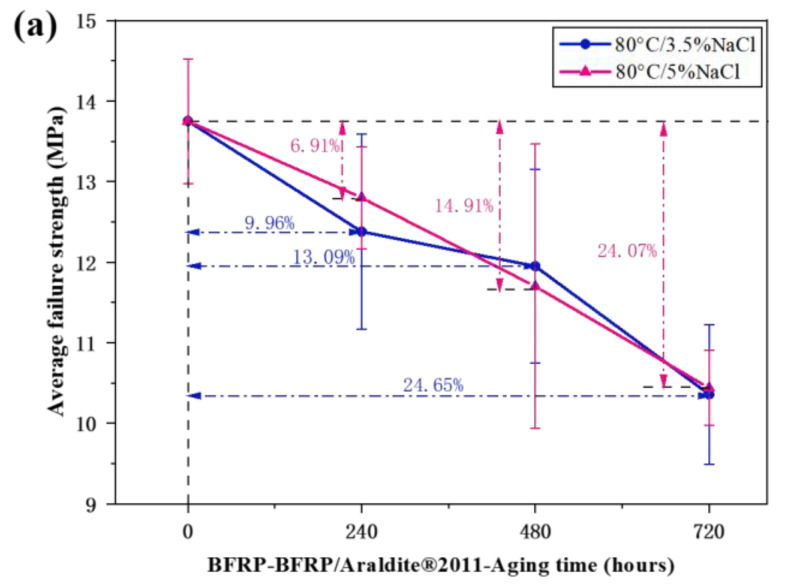

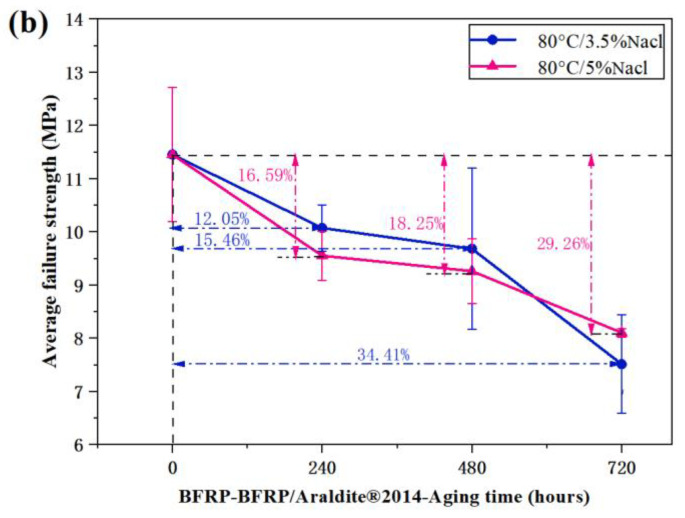
Diagrams of quasi-static shear strength: (**a**) Araldite^®^2011 adhesive joints, (**b**) Araldite^®^2014 adhesive joints.

After reaching the aging requirements, the joints of each group were subjected to the quasi-static tensile test. The experimental data of the mechanical properties of each group were processed and the average failure strength was calculated. The mechanical properties of the joints in different aging environments of the same adhesive were compared vertically with the data of the reference group, and the mechanical properties and aging characteristics of two adhesives with different properties in different aging environments were compared horizontally. The comparison results of the different aging environments of the two adhesives are shown in Figure 11.

Analysis of Figure 11a shows that for the Araldite^®^2011-bonded BFRP-BFRP joints, the effects of the two aging environments on the mechanical properties were roughly similar, and the overall trend was downward. After aging for 240 h, 480 h, and 720 h in the 80 °C/3.5% NaCl solution, the average ultimate failure strength of the joint was reduced by 9.96%, 13.09%, and 24.65%, respectively, compared to the unaged joint. In the whole aging process, the aging rate of the adhesive joint was the slowest in the period 240–480 h, the fastest in the period 480–720 h, and the aging rate first decreased and then increased with the increase in aging. In the environment of the 80 °C/5% NaCl solution, the joint was aged for 240 h, 480 h, and 720 h, and the average ultimate failure strength of the joint was reduced by 6.91%, 14.91%, and 24.07%, respectively, compared with the non-aged treatment. In this environment, the aging effect is significant and the rate of aging does not change significantly with increasing aging time. Comparing the two aging environments, when aging reaches 720 h, the mechanical properties of the joints under the two aging environments are similar.

By analyzing Figure 11b, it can be seen that for the Araldite^®^2014-bonded BFRP-BFRP joints, the effects of the 80 °C/3.5% NaCl solution and the 80 °C/5% NaCl solution on the mechanical properties of the aging environment were roughly similar, and the overall trend was downward. In the environment of the 80 °C/3.5% NaCl solution, aging for 240 h, 480 h, and 720 h, the average ultimate failure strength of the joint was reduced by 12.05%, 15.46%, and 34.41%, respectively, compared with the non-aging treatment. From the whole aging process, the aging rate of the adhesive joint was the slowest in the period 240–480 h, the fastest in the period 480–720 h, and the aging rate first decreased and then increased with the increase in aging. In the environment of the 80 °C/5% NaCl solution, the joint was aged for 240 h, 480 h, and 720 h, and the average ultimate failure strength of the joint was reduced by 16.59%, 18.25%, and 29.26%, respectively, compared to that without aging treatment. From the whole aging process, the aging rate of the adhesive joint was the slowest in the period 240–480 h, the aging rate was the fastest in the first 240 h, and the aging rate first decreased and then increased with the increase in aging. Comparing the two aging environments, it was found that the aging rate of the joint in the 80 °C/3.5% NaCl solution was slow before aging for 480 h, and the average ultimate failure strength only decreased by 15.46% when aged for 480 h, even less than the reduction in the average ultimate failure strength after aging for 240 h in the 80 °C/5% NaCl solution, but the joint would age rapidly in the period 480–720 h.

By combining Figure 11, it can be found that no matter what aging environment the BFRP joint is in, the aging degree of the mechanical properties of the joint is similar within the first 240 h of aging, which shows that the mechanical properties of BFRP joint materials decrease rapidly under the coupling of moisture and heat, and because the joint is still in the early stage of moisture absorption and deposition, the solution environment at this aging stage has no decisive relationship with aging failure. During the 240–480 h period of aging failure, the aging rate of the joint decreases in all environments, and water absorption tends to be saturated at this stage. When aging reaches 720 h, the aging degree of the two joints is similar, but the aging degree is greater in the 80 °C/3.5% NaCl solution, indicating that the reduction in mechanical properties of the joints is positively correlated with water concentration [58]. At the same time, comparing the two joints, it is not difficult to conclude that the 2011 joint is better than the 2014 joint in terms of joint strength, anti-aging performance, and aging rate stability.

### 3.5. Failure Displacement Analysis

**Figure 12:**
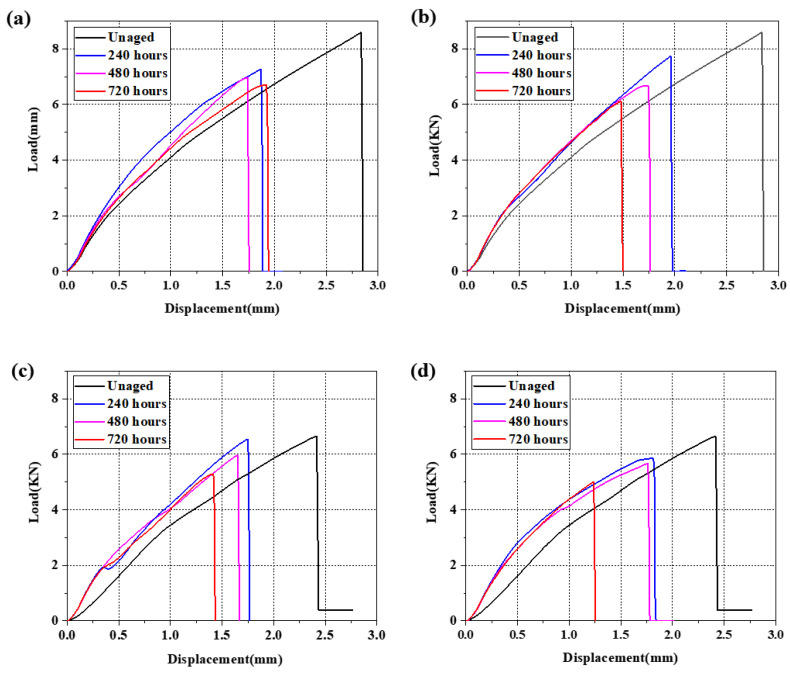
Load–displacement curves of two kinds of joints: (**a**) Araldite^®^2011 adhesive aged for 0/240/480/720 h in 3.5% NaCl environment; (**b**) Araldite^®^2011 adhesive aged for 0/240/480/720 h in 5% NaCl environment; (**c**) Araldite^®^2014 adhesive aged for 0/240/480/720 h in 3.5% NaCl environment; (**d**) Araldite^®^2014 adhesive aged for 0/240/480/720 h in 5% NaCl environment.

The load–displacement curves of each group of joints were obtained by tensile testing, as shown in Figure 12. Regardless of the aging environment or the properties of the adhesive, the end of the curve fluctuates in a zigzag pattern, i.e., there is a fairly sharp drop in the curve after the failure load is reached, indicating that the failure is a rapid final fracture [26], the maximum failure load and expiration displacement of the joint have a significant decreasing trend with aging. First, high temperatures will change the stress state of the joint, and the significant difference in thermal expansion coefficient will greatly change the stress–strain characteristics of the polymer adhesive and weaken the bonding strength [67]. At the same time, the temperatures above T_g_ softened the adhesive and resin matrix. Second, the wet–heat coupling will cause the cracking and debonding of the adhesive and matrix, and the fiber–matrix interface and interlayer delamination, further weakening the bonding interface and the fiber–matrix interlayer adhesion [29,30,31,32,33,34]. However, the curve of the sample aged for 240 h has a larger distance from the curve of the unaged sample and a smaller distance from the two subsequent aging curves; it can be concluded from above that the decreasing part of the curves is mainly concentrated in the early stage of the experiment, and the decreasing range is also basically decreasing. This is because the absorption of water is concentrated in the early stage of aging, and both the polymer matrix and the adhesive absorb water and expand, resulting in internal stress and partial hydrolysis, which not only reduces the glass transition temperature (T_g_), but also reduces the elastic modulus and strength of the adhesive and adhered materials [15]. In the early stage of aging, the slope of each curve increases slightly and then decreases slightly with aging, indicating that the stiffness of the joint after aging increases, but will recover with aging; the process is very slow. The increase in stiffness was proved to be due to the increase in secondary crosslinking between the bound water and the polymer chain promoted by the strong hygrothermal effect at the initial stage [68], and this structure would be destroyed by subsequent degradation or oxidation of the material.

The area under the load–displacement curve is the fracture energy of the joint. Its variation also gradually decreases with aging and the reduction amplitude decreases. Most of the reduction in the fracture energy also occurs in the early aging stage, corresponding to the change in failure load and failure displacement. From the two environments, the aging environment has little influence on the final failure load, failure displacement, and fracture energy reduction. Comparing the two kinds of adhesive, the mechanical properties of the 2011 joints are stronger than those of the 2014 joints both before and after aging, which is consistent with the results of the average failure strength curves.

### 3.6. Failure Mode Analysis

#### 3.6.1. Analysis of Failure Section Morphology

**Figure 13:**
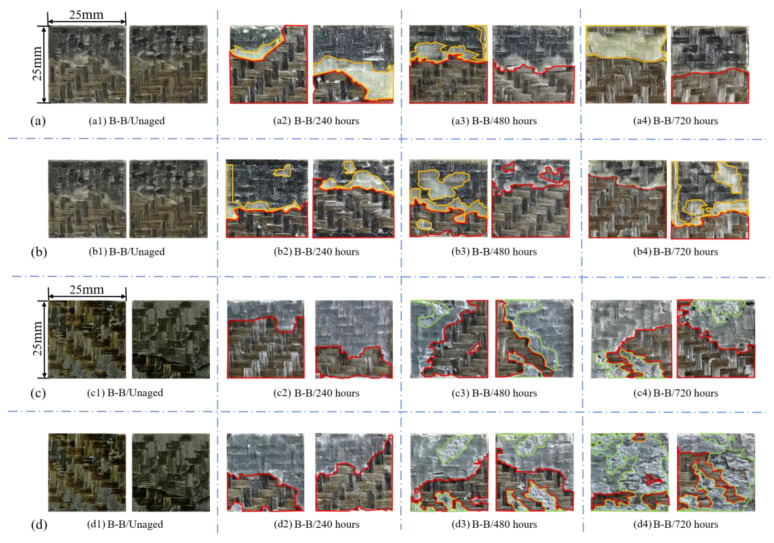
Macromorphology of two joint failure sections: (**a**: **a1**–**a4**) Araldite^®^2011 adhesive aged for 0/240/480/720 h in 3.5% NaCl environment; (**b**: **b1**–**b4**) Araldite^®^2011 adhesive aged for 0/240/480/720 h in 5% NaCl environment; (**c**: **c1**–**c4**) Araldite^®^2014 adhesive aged for 0/240/480/720 h in 3.5% NaCl environment; (**d**: **d1**–**d4**) Araldite^®^2014 adhesive aged for 0/240/480/720 h in 5% NaCl environment.

The most representative specimens of each group were selected to observe and analyze their failure modes. The macroscopic section morphology is shown in Figure 13.

It can be seen from Figure 13(a1,b1) that the failure modes of the unaged 2011 joints are all fiber tearing, such as the areas marked in red, which indicates that the joints have not made full use of the bonding properties of the adhesives. After aging for 240 h, the failure modes of Figure 13(a2,b2) are still dominated by fiber tearing, but cohesion failure of the adhesive layer fracture occurs at the same time, such as the areas marked in yellow. The reason for this is that when the temperature exceeds T_g_ it makes the adhesive soften, and in addition, after the adhesive absorbs water rapidly in the early stage of aging, water acts as a polar molecule and forms hydrogen bonds with hydroxyl groups, resulting in the destruction of the hydrogen bonds between the original molecular chain and increasing the length of the intersegment hydrogen bonds [69]. At the same time, hydrolysis leads to polymer chain fracture, decomposition of hard and side chains, reduction in molecular weight, increase in free volume, and increase in molecular mobility [70], all of which weaken the interchain forces of the polymer molecules and lead to the destruction of the adhesives. With the progression of aging, it can be found from Figure 13(a3,b3) that the failure mode after aging for 480 h is still dominated by fiber tearing. The results show that the bonding strength between fiber and matrix is weaker than the interface between adhesive and sheet due to the moisture and heat aging of BFRP. At 720 h, adhesive layer cohesion no longer stays at the bonding edge, but is distributed unevenly throughout the bonding region, because at this time the water molecules have completely opened the bond line and entered into the pores and cracks, and then continuously moved and reacted under the promotion of the high temperature, which further increases the porosity and degradation of the adhesive [71].

The change in failure mode of the 2014 joint is similar to that of the 2011 joint, but because the Araldite^®^2014 adhesive is brittle, the adhesive layer on the failure section is more uniform than that of the 2011 joint. It was observed that the failure section of the joint also became rough with obvious wrinkles with the increase in aging, which was caused by the plasticization of the adhesive. The plasticized adhesive extended in the fracture process of the sample, resulting in a relatively rough surface [72], and because its anti-aging property is weaker than that of the Araldite^®^2011 adhesive, the adhesive edge was seriously degraded and an irregular white edge appeared in direct contact with the water. DSC and failure strength analysis showed that the corrosion resistance of the Araldite^®^2014 adhesive was also inferior to that of the Araldite^®^2011 adhesive. Observation of the fracture appearance in the 2014 joints in salt solution showed that white patches appeared on the surface of the adhesive layer, as marked in green in the figure, in addition, the number of white patches increased with the increase in salt concentration and aging. The adhesive layer of the section aged for 720 h in 5% NaCl solution is almost covered with white patches, and these white patches are formed due to chemical corrosion of the adhesive caused by salt ions, which also leads to cracking due to reduced strength [39].

#### 3.6.2. Micromorphology Analysis

**Figure 14:**
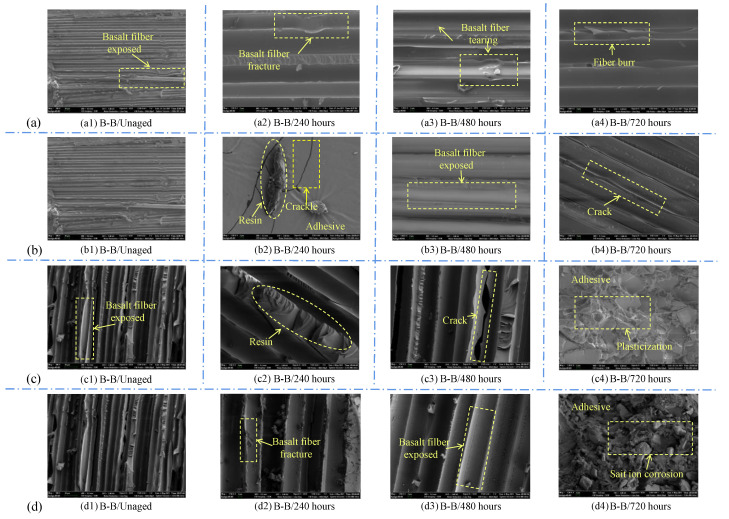
Two kinds of joint SEM scanning images: (**a**: **a1**–**a4**) Araldite^®^2011 adhesive aged for 0/240/480/720 h in 3.5% NaCl environment; (**b**: **b1**–**b4**) Araldite^®^2011 adhesive aged for 0/240/480/720 h in 5% NaCl environment; (**c**: **c1**–**c4**) Araldite^®^2014 adhesive aged for 0/240/480/720 h in 3.5% NaCl environment; (**d**: **d1**–**d4**) Araldite^®^2014 adhesive aged for 0/240/480/720 h in 5% NaCl environment.

Combined with the above failure mode analysis, SEM scanning was carried out on the areas with obvious section characteristics, and the microscopic morphology was collected under the acceleration voltage of 5.0 kV and working distance of 5 mm, as shown in Figure 14.

The filaments exposed after tearing of the BFRP can be clearly seen from the label in the unaged sample (a1). Such a situation also exists in the other figures, which is consistent with the previous judgment of the failure mode. In addition to the torn part of the fiber in the labeled area in (a3), there is also fiber deformation failure in the labeled area in (a2) or serrated burrs on the surface of the fiber in (a4). (b2) is a point on the adhesive layer, although the surface is relatively smooth, but it is covered with cracks, while the residual resin is torn, indicating that the adhesive and resin matrix have suffered a certain degree of damage at this point. In addition, obvious cracks can be seen in the marked area in (b4). Such cracks often deposit more water, and the irregular movement of water at high temperatures increases the size of the pores, resulting in the formation of cracks under the action of load. The marked area in (c4) is in sharp contrast to the other parts, and the plasticizing effect will make the rubber layer’s cross-section rough and form a ridge. The 2014 joint produced white spots after aging in salt solution for a long time. The SEM image of this joint corresponds to the marked area in (d4), and it can be seen that chemical corrosion due to salt ions in this area has caused dense pits in the otherwise more continuous adhesive layer after brittle fracture, which is also reflected in (c4), but it is not as obvious as that in the 5% NaCl solution.

**Figure 15:**
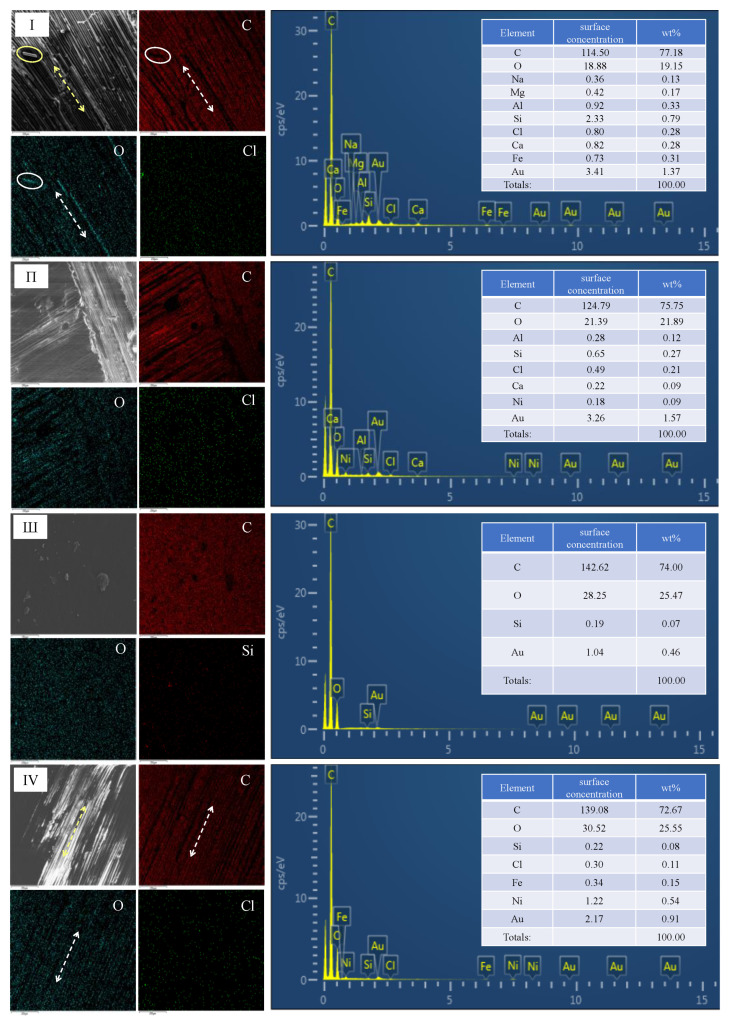
EDX image of Araldite^®^2011 adhesive joint fracture surface: (**I**) aged for 240 h in 3.5% NaCl environment; (**II**) aged for 720 h in 3.5% NaCl environment; (**III**) aged for 240 h in 5% NaCl environment; (**IV**) aged for 720 h in 5% NaCl environment.

Due to the better performance of the 2011 adhesive joint, EDX analysis was carried out on the characteristic areas of the joint aged for 240 h and 720 h under two conditions, and the failure mechanism of the joint was summarized from the aspect of element changes. The electronically scanned images in Figure 15 are all taken from the well featured areas in Figure 13. From Figure 15(I/IV), the exposed fiber filaments and resin matrix can be clearly observed. By analyzing the atlas and data of the elements C and O, it can be found that the concentration of these two elements is high, and that O has obvious traces of distribution along the fiber, while the distribution position of C is just opposite to that of O, and there is almost no C at the exposed part of the fiber, indicating that O mainly exists on the fiber, while C mainly exists on the resin matrix. The O/C ratio reflects the degree of hydrolysis of the material. This ratio increases from 0.25 to 0.29 from (I) to (II), and the ratio from (III) to (IV) increases from 0.34 to 0.35, indicating that the degree of hydrolysis increases with aging. The uniform distribution of Cl on the section indicates that chloride ions have been diffused throughout the joint at 720 h.

## 4. Conclusions

In this paper, the durability of BFRP-BFRP single-lap joints bonded with two types of adhesives dipped in 80 °C seawater was studied. The experiment was divided into groups according to the adhesive, aging environment, and aging. The water absorption test results of the joint materials were fitted by a sequential dual Fickian model, and the glass transition temperature (T_g_) of each group of adhesives was determined by DSC. The thermal stability properties of the two adhesives were compared using TGA-DTG curves, and the mechanical properties of the joints of each group were measured using the quasi-static tensile test. The complete failure process was described by the load–displacement curves. Finally, the evolution of the failure mode during the failure process was investigated by combining the macro cross-sections and SEM-EDX microscopic morphology. To provide reference data for the safety of BFRPs applied in shipbuilding, based on the experimental results, the following conclusions are drawn:

(1) The water absorption of materials can be fitted closely by the sequential dual Fickian (SDF) model. The first stage of water absorption is mainly by diffusion, then, salt ion corrosion and the formation of bound water leads to the second stage of water absorption. The hydrophilicity of the Araldite^®^2011 adhesive is the strongest, followed by the Araldite^®^2014 adhesive, BFRP plate is the smallest, so hydrolysis mainly occurs on the adhesive.

(2) The decrease in T_g_ of the adhesive is related to plasticization and a decrease in the crosslinking density. The overall decrease in T_g_ of the Araldite^®^2011 adhesive with stronger hydrophilicity is smaller than that of the Araldite^®^2014 adhesive due to the formation of hydrogen bonds by the binding water.

(3) The aging environment and aging have no significant influence on the thermal stability of the adhesives. The two adhesives exhibit the same third-order weight loss in the process of thermal weight loss due to their similar polymer properties. Based on the final weight loss rate, the thermal stability of the Araldite^®^2014 adhesive is stronger than that of the Araldite^®^2011 adhesive.

(4) According to the analysis of the average failure strength of the joint, the decrease in mechanical properties promoted by temperatures of 80 °C is positively correlated with the water concentration, but the chemical corrosion is also enhanced with the increase in salt ion concentration. The 2011 joints showed better aging resistance and stability in both environments, which is consistent with the results obtained from the DSC tests.

(5) The failure of the joint is a rapid ultimate fracture, which is an outbreak disaster caused by a series of adverse changes such as thermal expansion stress, hydrolysis, T_g_ reduction, degradation of the adhesive and BFRP resin matrix, bond interface debonding, and so on. The crosslinking of bound water increases the joint stiffness, but this structure will be slowly destroyed with aging.

(6) The failure mode of the joint changes from single fiber tear to fiber tear and adhesive layer cohesion with aging, because the bonding property of the hydrolyzed adhesive cannot be fully utilized. The bonding edge in direct contact with water suffers the most serious damage, this is also where the adhesive cracking first occurs. After a long period of aging, the chemical corrosion of salt ions will obviously destroy the rubber layer, and the cross-section will become rough due to the plasticizing effect.

## Figures and Tables

**Table 1:** Properties of epoxy resin and unidirectional fabric.

ML-5417A /ML-5417B Epoxy Resin		Basalt Fiber Unidirectional Fabric	
Cure condition	25 °C × 24 h + 100 °C × 3 h	Surface Density/(g·cm^−2^)	300
		Tensile strength/(MPa)	2100
Epoxy value/(g/ep)	165–175	Young's modulus/(GPa)	105
25 °C Density/(g·cm^−3^)	1.10–1.20	Elongation/(%)	2.6
Tensile modulus/(MPa)	2800–3200	Nominal thickness/(mm)	0.115
T_g_/(°C)	110–125	Single fiber size/(μm)	13

**Table 2:** Properties of Araldite^®^2011 and Araldite^®^2014.

	Araldite^®^2011	Araldite^®^2014
Young’s modulus, E (GPa)	1.65	4
Shear modulus, G (GPa)	0.2	1.2
Density (kg·m^3^)	1.15	1.6
Poisson’s ratio	0.43	0.33

**Table 3:** Main weight loss zone ranges, maximum weight loss rate temperatures, and residual rates of two kinds of adhesive TGA-DTG curves.

Adhesive	Environment	Aging Time (Hours)	Primary Pyrolysis Initial Temperature (°C)	Maximum Weight Loss Rate Temperature (°C)	Residue Rate at 800 °C (%)
Araldite^®^2011	Unaged	0	251.6–513.3	430.8	1.36%
	3.5% NaCl	240	260.8–521.6	425	5.35%
		480		427.5	3.31%
		720		423.3	4.73%
	5% NaCl	240	265.7–511.7	421.7	3.54%
		480		424.1	3.51%
		720		422.5	2.15%
Araldite^®^2014	Unaged	0	245.8–514.2	361.7	40.97%
	3.5% NaCl	240	241.7–503.5	356.7	40.72%
		480		360.8	41.01%
		720		362.5	39.11%
	5% NaCl	240	240.8–501.7	359.1	41.08%
		480		358.3	41.6%
		720		360.6	41.72%

## Data Availability

The data presented in this study are available on request from the corresponding author.

## References

[1] Wang K., Lu Y., Rao Y., Wei N., Ban J., Peng Y., Yao S., Ahzi S. (2020). New insights into the synergistic influence of voids and interphase characteristics on effective properties of unidirectional composites. Compos. Struct..

[2] Lin J., Sun C., Min J., Wan H., Wang S. (2020). Effect of atmospheric pressure plasma treatment on surface physicochemical properties of carbon fiber reinforced polymer and its interfacial bonding strength with adhesive. Compos. Part B Eng..

[3] Zhou A., Qin R., Chow C.L., Lau D. (2020). Bond integrity of aramid, basalt and carbon fiber reinforced polymer bonded wood composites at elevated temperature. Compos. Struct..

[4] Cui J., Gao S., Jiang H., Huang X., Lu G., Li G. (2020). Adhesive bond-electromagnetic rivet hybrid joining technique for CFRP/Al structure: Process, design and property. Compos. Struct..

[5] Duan S., Zhang Z., Wei K., Wang F., Han X. (2020). Theoretical study and physical tests of circular hole-edge stress concentration in long glass fiber reinforced polypropylene composite. Compos. Struct..

[6] Liu S., Yang T., Liu C., Jin Y., Sun D., Shen Y. (2020). Modelling and experimental validation on drilling delamination of aramid fiber reinforced plastic composites. Compos. Struct..

[7] Altalmas A., Refai A.E., Abed F. (2015). Bond degradation of basalt fiber-reinforced polymer (BFRP) bars exposed to accelerated aging conditions. Constr. Build. Mater..

[8] Fiore V., Scalici T., Bella G.D., Valenza A. (2015). A review on basalt fifibre and its composites. Compos. Part B Eng..

[9] Nassiraei H., Rezadoost P. (2021). SCFs in tubular X-connections retrofitted with FRP under in-plane bending load. Compos. Struct..

[10] Nassiraei H., Rezadoost P. (2021). SCFs in tubular X-joints retrofitted with FRP under out-of-plane bending moment. Mar. Struct..

[11] Li J., Li Y., Xiang Y., Pan Q., Chen C., Liu J., Hu X. (2020). Effect of hygrothermal-mechanical exposure on the residual strength of adhesively bonded joints. Int. J. Adhes. Adhes..

[12] Gonilha J.A., Barros J., Correia J.R., Sena-Cruz J., Branco F.A., Ramos L.F., Gonçalves D., Alvim M.R., Santos T. (2014). Static, dynamic and creep behavior of a full-scale GFRP-SFRSCC hybrid footbridge. Compos. Struct..

[13] Keller T., Theodorou N.A., Vassilopoulos A.P., Castro J.D. (2015). Effect of natural weathering on durability of pultruded glass fiber–reinforced bridge and building structures. J. Compos. Constr..

[14] Jingxin N., Wenlong M., Guofeng Q., Wei T., Leixin P. (2018). Effect of Temperature on the Mechanical Properties of Adhesively Bonded Basalt frp-aluminum Alloy Joints in the Automotive Industry. Int. J. Adhes. Adhes..

[15] Sousa J.M., Correia J.R., Gonilha J., Cabral-Fonseca S., Firmo J.P., Keller T. (2019). Durability of adhesively bonded joints between pultruded GFRP adherends under hygrothermal and natural ageing. Compos. Part B Eng..

[16] Chen Y., Yang X., Li M., Wei K., Li S. (2019). Mechanical behavior and progressive failure analysis of riveted, bonded and hybrid joints with CFRP-aluminum dissimilar materials. Thin Wall Struct..

[17] Li H., Zhang K., Fan X., Cheng H., Xu G., Suo H. (2019). Effect of seawater ageing with different temperatures and concentrations on static/dynamic mechanical properties of carbon fiber reinforced polymer composites. Compos. Part B Eng..

[18] Mouritz A.P., Gellert E., Burchill P., Challis K. (2001). Review of advanced composite structures for naval ships and submarines. Compos. Struct..

[19] Tran P., Nguyen Q.T., Lau K.T. (2018). Fire performance of polymer-based composites for maritime infrastructure. Composites.

[20] Jianze L., Jingxin N., Wei T., Wenlong M., Guangbin W., Yuan G. (2021). Comparative study on mechanical properties of aluminum alloy and BFRP single lap joints with hygrothermal aging. J. Adhes..

[21] Ray B.C., Rathore D. (2015). Environmental damage and degradation of FRP composites: A review report. Polym. Compos..

[22] Zhang F., Wang H.P., Hicks C., Carlson B.E., Yang X., Zhou Q. Effect of Prelube, Surface Coating and Substrate Materials on Initial Strength of Adhesive Joints Between Al Alloy and Steels. Proceedings of the ASME 2011 International Mechanical Engineering Congress and Exposition.

[23] Zhang F., Yang X., Wang H.P., Zhang X., Xia Y., Zhou Q. (2013). Durability of Adhesively bonded Single Lap–Shear Joints in Accelerated Hygrothermal Exposure for Automotive Applications. Int. J. Adhes. Adhes..

[24] Avendaño R., Carbas R.J.C., Marques E.A.S., Da Silva L.F.M., Fernandes A.A. (2016). Effect of temperature and strain rate on single lap joints with dissimilar lightweight adherends bonded with an acrylic adhesive. Compos. Struct..

[25] Banea M.D., da Silva L.F.M. (2010). The effect of temperature on the mechanical properties of adhesives for the automotive industry. J. Mater. Des. Appl..

[26] Yao M., Zhu D., Yao Y., Zhang H., Mobasher B. (2016). Experimental study on basalt FRP/steel single-lap joints under different loading rates and temperatures. Compos. Struct..

[27] Chen Y., Li M., Yang X., Wei K. (2020). Durability and mechanical behavior of CFRP/Al structural joints in accelerated cyclic corrosion environments. Int. J. Adhes. Adhes..

[28] Budhe S., Banea M.D., Barros S.D., Silva L.F.M.D. (2017). An updated review of adhesively bonded joints in composite materials. Int. J. Adhes. Adhes..

[29] Gautier L., Mortaigne B., Bellenger V. (1999). Interface damage study of hydrothermally aged glass-fibre-reinforced polyester composites. Compos. Sci. Technol..

[30] Arun K.V., Basavarajappa S., Sherigare B.S. (2010). Damage characterization of glass/textile fabric polymer hybrid composites in sea water environment. Mater. Des..

[31] Regazzi A., Corn S., Ienny P., Benezet J.-C., Bergeret A. (2016). Reversible and irreversible changes in physical and mechanical properties of bio-composites during hydrothermal aging. Ind. Crops Prod..

[32] Dhakal H.N., Zhang Z.Y., Richardon M.O.W. (2007). Effect of water absorption on the mechanical properties of hemp fibre reinforced unsaturated polymer composites. Compos. Sci. Technol..

[33] Kaelble D.H., Dynes P.J., Crane L.W. (1975). Interfacial mechanisms of moisture degradation in graphite-epoxy composites. J. Adhes..

[34] Deneve B., Shanahan M.E.R. (1995). Physical and chemical effects in an epoxy-resin exposed to water-vapor. J. Adhes..

[35] Banea M.D., da Silva L.F.M. (2009). Adhesively bonded joints in composite materials: An overview. J. Mater. Des. Appl..

[36] Heshmati M., Haghani R., Al-Emrani M. (2015). Environmental durability of adhesively bonded FRP/steel joints in civil engineering applications: State of the art. Compos. B Eng..

[37] Cabral-Fonseca S., Correia J.R., Rodrigues M.P., Branco F.A. (2012). Artificial accelerated ageing of GFRP pultruded profiles made of polyester and vinylester resins: Characterisation of physical-chemical and mechanical damage. Strain.

[38] Banea M.D., Silva L.F.M., Carbas R.J.C., Barbosa A.Q., De Barros S., Viana G. (2018). Effect of water on the behavior of adhesives modified with thermally expandable particles. Int. J. Adhes. Adhes..

[39] Li S., Guo S., Yao Y., Jin Z., Shi C., Zhu D. (2021). The effects of aging in seawater and SWSSC and strain rate on the tensile performance of GFRP/BFRP composites: A critical review. Constr. Build. Mater..

[40] Zhang Y., Vassilopoulos A.P., Keller T. (2009). Environmental effects on fatigue behavior of adhesively-bonded pultruded structural joints. Compos. Sci. Technol..

[41] Kootsookos A., Mouritz A.P. (2004). Seawater durability of glass- and carbon-polymer composites. Compos. Sci. Technol..

[42] Wei B., Cao H., Song S. (2011). Degradation of basalt fiber and glass fiber/epoxy resin composites in seawater. Corros. Sci..

[43] Mourad A.H.I., Idrisi A.H., Wrage M.C., Beckry M.A. (2019). Long-term durability of thermoset composites in seawater environment. Compos. B Eng..

[44] Wang X., Zhao X., Wu Z. (2018). Fatigue degradation and life prediction of basalt fiber-reinforced polymer composites after saltwater corrosion. Mater. Des..

[45] Wang Y.L., Guo X.Y., Shu S.Y.H., Guo Y.C., Qin X.M. (2020). Effect of salt solution wet-dry cycling on the bond behavior of FRP-concrete interface. Constr. Build. Mater..

[46] Lu Z., Li Y., Xie J. (2020). Durability of BFRP bars wrapped in seawater sea sand concrete—Science Direct. Compos. Struct..

[47] Nian X.I. (2000). Damage characterization and failure analysis in fifiber reinforced composites. J. Aeronaut. Mater..

[48] Li J., Yan Y., Zhang T., Liang Z. (2015). Experimental study of adhesively bonded CFRP joints subjected to tensile loads. Int. J. Adhes. Adhes..

[49] (2014). Standard Test Method for Lap Shear Adhesion for Fiber Reinforced Plastic (FRP) Bonding.

[50] Zaeri A.R., Saeidi Googarchin H. (2019). Experimental investigation on environmental degradation of automotive mixed-adhesive joints. Int. J. Adhes. Adhes..

[51] Ekrem M., Avcı A. (2018). Effects of polyvinyl alcohol nanofiber mats on the adhesion strength and fracture toughness of epoxy adhesive joints. Compos. Part B Eng..

[52] Aghamohammadi H., Hosseini Abbandanak S.N., Eslami-Farsani R., Siadati S.M.H. (2018). Effects of various aluminum surface treatments on the basalt fiber metal laminates interlaminar adhesion. Int. J. Adhes. Adhes..

[53] Marques G.P., Campilho R.D.S.G., da Silva F.J.G., Moreira R.D.F. (2016). Adhesive selection for hybrid spot-welded/bonded single-lap joints: Experimentation and numerical analysis. Compos. Part. B-Eng..

[54] (2003). Adhesives-Determination of Tensile Lap-Shear Strength of Rigid-to-Rigid Bonded Assemblies.

[55] Jadhav N.R., Gaikwad V.L., Nair K.J., Kadam H.M. (2009). Glass transition temperature: Basics and application in pharmaceutical sector. Asian J. Pharm..

[56] Mu W., Qin G., Na J., Tan W., Liu H., Luan J. (2019). Effect of alternating load on the residual strength of environmentally aged adhesively bonded CFRP-aluminum alloy joints. Compos. Part B Eng..

[57] Lin Y.C., Chen X. (2005). Moisture sorption–desorption–resorption characteristics and its effect on the mechanical behavior of the epoxy system. Polymer.

[58] Ameli A., Datla N.V., Papini M., Spelt J.K. (2010). Hygrothermal Properties of Highly Toughened Epoxy Adhesives. J. Adhes..

[59] LaPlante G., Ouriadov A.V., Lee-Sullivan P., Balcom B.J. (2008). Anomalous moisture diffusion in an epoxy adhesive detected by magnetic resonance imaging. J. Appl. Polym. Sci..

[60] Han X., Pickering E., Bo A., Gu Y. (2020). Characterisation on the hygrothermal degradation in the mechanical property of structural adhesive: A novel meso-scale approach. Compos. Part B Eng..

[61] Emara M., Torres L., Baena M., Barris C., Moawad M. (2017). Effect of sustained loading and environmental conditions on the creep behavior of an epoxy adhesive for concrete structures strengthened with CFRP laminates. Compos. Part B Eng..

[62] (1999). Standard Test Methods of Polymers by Differential Scanning Calorimetry.

[63] Lin Y.C., Chen X., Zhang H.J., Wang Z.P. (2006). Effects of hygrothermal aging on epoxy-based anisotropic conductive film. Mater. Lett..

[64] Galvez P., Abenojar J., Martinez M.A. (2019). Effect of moisture and temperature on the thermal and mechanical properties of a ductile epoxy adhesive for use in steel structures reinforced with CFRP. Composites.

[65] Vieira P., Souza F., Cardoso D., Vieira J.D., de Andrade Silva F. (2020). Influence of moderate/high temperatures on the residual flexural behavior of pultruded GFRP. Compos. Part B Eng..

[66] Jiang X., Li C., Chi Y., Yan J. (2010). TG-FTIR study on urea-formaldehyde resin residue during pyrolysis and combustion. J. Hazard. Mater..

[67] Adams R.D., Coppendale J., Mallick V., Al-Hamdan H. (1992). The effffect of temperature on the strength of adhesive joints. Int. J. Adhes. Adhes..

[68] Galvez P., Abenojar J., Martinez M.A. (2019). Durability of steel-CFRP structural adhesive joints with polyurethane adhesives. Compos. B Eng..

[69] Nachtane M., Tarfaoui M., Sonia S., Moumen A.E., Saifaoui D. (2019). An investigation of hygrothermal aging effects on High Strain Rate Behaviour of Adhesively Bonded Composite Joints. Compos. Part. B Eng..

[70] Fan Y., Na J., Mu W., Qin G., Tan W. (2019). Effect of Hygrothermal Cycle Aging on the Mechanical Behavior of Single-lap Adhesive Bonded Joints. J. Wuhan Univ. Technol. Mater. Sci. Ed..

[71] Merdas I., Thominette F., Teharkhtchi A., Verdu J. (2002). Factors governing water absorption by composite matrices. Compos. Sci. Technol..

[72] Liu S., Cheng X., Zhang Q., Zhang J., Bao J., Guo X. (2016). An investigation of hygrothermal effects on adhesive materials and double lap shear joints of CFRP composite laminates. Compos. B Eng..

